# A Novel ST-YOLO Network for Steel-Surface-Defect Detection

**DOI:** 10.3390/s23229152

**Published:** 2023-11-13

**Authors:** Hongtao Ma, Zhisheng Zhang, Junai Zhao

**Affiliations:** 1 School of Mechanical Engineering, Southeast University, Nanjing 211189, China; oldbc@seu.edu.cn; 2 College of Marine Electrical and Intelligent Engineering, Jiangsu Maritime Institute, Nanjing 211100, China; zhaojunai80@163.com

**Keywords:** artificial intelligence, surface-defect detection, object detection, YOLO Network

## Abstract

Recent progress has been made in defect detection using methods based on deep learning, but there are still formidable obstacles. Defect images have rich semantic levels and diverse morphological features, and the model is dynamically changing due to ongoing learning. In response to these issues, this article proposes a shunt feature fusion model (ST-YOLO) for steel-defect detection, which uses a split feature network structure and a self-correcting transmission allocation method for training. The network structure is designed to specialize the process of classification and localization tasks for different computing needs. By using the self-correction criteria of adaptive sampling and dynamic label allocation, more sufficiently high-quality samples are utilized to adjust data distribution and optimize the training process. Our model achieved better performance on the NEU-DET datasets and the GC10-DET datasets and was validated to exhibit excellent performance.

## 1. Introduction

Steel sheets are a fundamental component of many manufactured goods and hence must be of the highest quality. Imperfections in the steel sheet lower its aesthetic value and shorten its lifespan [1]. Steel-defect detection is conducted to make sure the final product is of high quality and to find out what went wrong. Metal sheets play a very important role in the field of mechanical processing and are indispensable raw materials. The surface quality of metal sheets is an important standard that determines their price. Due to limitations in current equipment and process conditions, different forms and types of defects are inevitably generated on the surface of metal plates, and the resulting defects vary greatly in size and quantity. Common defects include mesh patterns, inclusions, markings, surface pitting, iron oxide skin indentation, scratches, etc. Surface defects often become the starting point of metal corrosion. The presence of surface defects on steel plates can greatly reduce the fatigue strength of parts, affect the performance and lifespan of machines and instruments, and ultimately affect the performance and quality of products. Therefore, timely detection of small defects on the surface of steel plates and evaluation of the severity of steel-plate quality are of great significance for improving the surface quality and direct economic benefits of metal steel plates. With the application of computer vision in automated optical inspection (AOI), the problems of high labor costs, strong subjective interference, and low inspection efficiency of manual inspection have been solved, and the accuracy and time of inspection have been improved [2].

Defect detection is typically broken down into two stages: locating the problem and quantifying its severity. Both are analogous to computer vision classification and localization tasks [3]. Metal surface flaws can be discovered with the help of image processing. The recognition of defects and their type is critical to the analysis of process problems, and the precise position and area percentage of defects are required for quantitative evaluation of product surface quality.

Traditional methods based on manually designed features, commonly utilize a pipeline consisting of a series of components to perform defect detection. The process generally includes image pre-processing, region of interest (RoI) searching, feature extraction, feature selection, and pattern recognition. In most of the work, feature extraction relies on hand-crafted features, including approaches such as statistical methods [4,5], spectral methods [6,7], and model-based methods [8,9]. However, the approaches rely on a great deal of prior knowledge and design experience from experts [10]. In addition, the components of traditional methods are relatively independent of each other and it is difficult to achieve global optimization in an end-to-end manner.

Convolutional-neural-network architectures are widely used in deep learning methods for defect identification because of their ability to automatically learn features that interpret images. It optimizes feature extraction, utilizing a gradient update during training, which allows for more precise feature calculation. The fact that it is data-driven means that it may be used in a variety of contexts and is resilient to a wide range of material surfaces and defect types. The methods have come a long way in terms of speed and accuracy of flaw detection in recent years [11].

**Figure 1:**
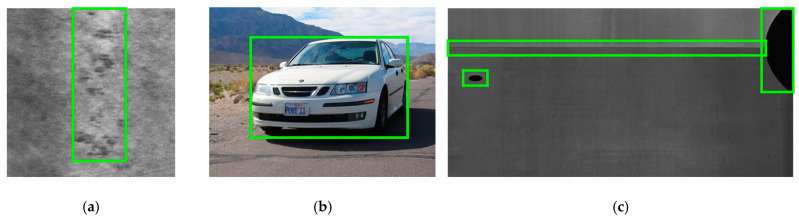
Images of different objects: (**a**) defect of rolled-in scale without a closed outline, (**b**) car with a clear outline in the natural scene, and (**c**) different defects of various shapes and various sizes.

Object detection algorithms are widely employed in defect detection [12] due to their low cost of annotation and high discrimination precision. In most cases, deep learning models are used in the approaches to pinpoint problem areas and classify defects. But current methods are not without their own set of problems. Firstly, most general object detectors are designed for the detection task of natural scenes; unlike natural objects with clear outlines, some discrete defects do not have closed overall outlines in the image, as compared in Figure 1a,b. Secondly, the image features of defects are rich in hierarchical levels, and the low-level features also play a critical role [13], which are not well used in most general detectors. Therefore, the model structure of general object detectors is not suitable enough for processing industrial images. Improved usage of semantic features across several layers and model structure optimization for learning classification and localization is also necessary for defect detectors. Third, as shown in Figure 1c, flaws can be discovered in a wide range of sizes and forms, making it impossible for a single sampling technique to account for all of them. Finally, the feature description complexity of different defects is various, and the number and quality of the corresponding adaptable samples generated from the model are also diverse. Moreover, with the progress of model training, the fitting degree of the model to the data, is increasing gradually; thus, the selection criteria of the samples also need to be changed accordingly. Therefore, a dynamic training algorithm with high flexibility is required to supervise the training of the model to adapt to the changeable data distribution of defects.

This work proposes a model for steel-surface-defect identification called ST-YOLO to address the aforementioned issues. Our model employs a shunt feature fusion network topology inspired by YOLOX [14] and trains itself with an adaptive core prior to producing accurate label assignments. The main contributions of this paper are as follows:

(1) To accomplish classification and localization, a shunt feature fusion network has been developed. The network’s fitting capability is optimized by tailoring the calculation methods for each task to the varying semantic levels of the two tasks.

(2) Adaptive core prior is proposed to suit the defects of different shapes, flexibly extracting the sample points.

(3) The self-tuning label assignment strategy is established. Based on the adaptive core prior, the strategy selects high-quality training samples using dynamic criteria involving category and location fitness, and then assigns appropriate target labels for the selected samples.

The rest of this paper proceeds as follows: The newest developments in object detection methods and the related field of defect detection are presented in Section 2. The proposed model framework and method are presented in Section 3. In Section 4, we undertake an experiment to evaluate ST-YOLO and compare the results to those obtained using alternative models. In Section 5, we show how the network structure and training mechanism actually work. Section 6 provides the final analysis.

## 2. Related Work

### 2.1. Deep-Learning-Based Defect Detection

Detection methods based on deep learning, process the images using learned features instead of designed features, contributing to outstanding performance in automatic defect detection.

Among the inspection methods based on deep learning, the research on anchor-based object detectors is relatively well-developed. In most cases, anchor boxes are used to indicate where flaws are located inside the defect region, which is considered the detection object. Cascade heads were used in Faster R-CNN by Liu et al. [15] to detect surface flaws in metal. Cheng et al. [16], building on RetinaNet, fused features using a channel attention mechanism and ASFF to identify flaws in steel surfaces. Ma et al. [17] improved the speed of YOLOv4 by adding an attention module.

However, anchor-based object detectors are limited by the prior anchor configuration; thus, more and more studies prefer anchor-free detectors. The anchor-free mechanism does not require prior boxes manually designed in advance, which helps to achieve end-to-end applications. Moreover, without generating a great number of dense candidate boxes, the detection speed can be improved. Kou et al. [18] transformed YOLOv3 into an anchor-free model, which increased the detection speed of strip defects. To better detect steel-surface flaws, Tian et al. [19] presented DCC-CenterNet, which uses dilated convolution to improve features and center weight to increase heatmaps. To lessen the dropout of imperfect features during fusion, Yu et al. [20] of the FCOS-based work proposed a bidirectional feature fusion network.

Unfortunately, there are still shortcomings in the existing approaches. First, some defects (such as cracks and spots) with ambiguous boundaries bring trouble for the model in determining the region of such defects, but little research has been undertaken on the model structure to improve the feature learning process of such defects. Second, the diversity of defects leads to large variations in the data distribution, which is still not solved by end-to-end learning in most of the works. Third, the training samples of the model change with the training process, but most algorithms adopt static training rules, which will limit the learning ability of the model.

### 2.2. Advances in Object Detection

The object detection method for region-level recognition can recognize the object in the image, simultaneously detecting the category, location (including position and size), and other information of the objects [21]. Classification and localization are the two tasks of object detection, and their fitting processes are similar but different in some aspects. Early object detectors such as Faster R-CNN [22], DCN [23], YOLOv3 [24], etc., shared the calculation course in the feature extraction and feature fusion stages, and the classification and localization results were not calculated separately until the last layer of the detection head. In recent years, some works [25,26,27] have re-concentrated the dual-task divergence, replacing sibling heads with parallel heads based on shared features, such as FCOS [28], FoveaBox [29], TSD [25], etc. Because the features that the two tasks are sensitive to are quite different, the features need to be processed differently. The separately computing network design allows the running of dual tasks to optimize their respective sub-networks during training, which means reducing the interference of coupling optimization. Furthermore, the features of interest can be processed individually during inference in the network structure.

With the emergence of anchor-free detectors, the pattern of the assignment of training labels is starting to attract attention. The dense prediction method widely used usually divides candidate samples into positive and negative samples and then matches the target labels for the positive samples to complete the training. Therefore, the training strategy will determine the upper limit of the model performance. To automatically separate training samples into positive and negative subsamples based on statistical features, Zhang et al. [30] suggested an adaptive training sample selection (ATSS) technique. Different from traditional detectors such as RetinaNet [31], which adopt a predetermined fixed threshold of IoU (Intersection over Union value) to filter positives, ATSS determines the dynamic threshold through the overall IoU statistics. In addition, some algorithms are comprehensively designed for co-optimization of classification and localization. Kim et al. [32] proposed a probabilistic anchor assignment method (PAA) to fit the distribution of the training loss of candidates, using a Gaussian mixture model to determine a threshold to distinguish positive and negative anchors. Ge et al. [33] proposed an optimal-transport-based assignment method (OTA), which calculates the sum cost of classification and regression and then treats the assignment as an optimal transport problem. By adjusting the threshold, these methods help the model to accustom to the appropriate matching strategy for different data distributions and different training stages. At the same time, the limitation of hyperparameters on training is greatly reduced.

This paper takes YOLOX as the baseline, which adopted decoupled head and training methods such as multiple positive sampling and a simplified OTA method (SimOTA), which achieves a high level of accuracy and speed. Based on YOLOX, we further propose a shunt network architecture for a dual-task and self-tuning training strategy for label assignment, which will be applied to surface-defect detection.

## 3. Methodology

**Figure 2:**
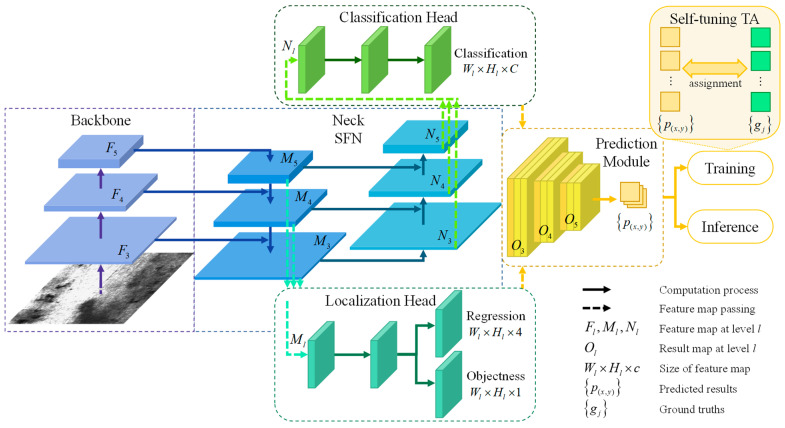
Detailed structure of proposed ST-YOLO.

Figure 2 provides an overview of the ST-YOLO architecture. First, the picture features are extracted via the CSPDarkNet53 [34] backbone network, and the feature maps are withdrawn at various stages. The shunt fusion network (SFN) then uses separate fusion flows to produce localization and classification feature pyramids. Finally, we aggregate the results of classification, regression, and objectness to derive the predictions for each location. To train a network, we first apply self-tuning transport assignment (STTA) to pair up predicted samples with their correct labels, and then we compute the loss of positive samples, and finally we backpropagate the gradient to update the network. Defect categories and confidence scores are anticipated after the inference process has completed post-processing, which may include non-maximum suppression (NMS).

In the neck region, our model is an improvement over the baseline. Additionally, STTA enhanced on SimOTA allows for synchronous adjustments to sample criteria based on the geometry of the GT and the degree of difficulty in fitting.

### 3.1. Shunt Fusion Network

Categories and locations have distinct levels of meaning, which creates the problem of incompatible computing needs. Even if features are calculated separately in each brain with the default YOLOX, the same feature map is being fed into both heads. Since the features provided to the dual tasks are identical at the semantic level, the baseline does not perform a full differential computation. To address this shortcoming, we generate distinct feature maps for classification and localization in the model’s head.

The semantic level of the final stage of fusion in the feature fusion network is higher than that of the initial stage. As can be seen in Figure 2, the fusion uses a top-down approach in the localization sub-branch. The localization head receives the combined features from the backbone network and the deeper characteristics. Some examples of primitive features are texture, edge, shape, and so on, all of which rely on less-detailed information. Using Equations (1) and (2), we can derive the feature map. Bottom-up information flow characterizes the fusion mode in the classification subfield. Deep features are created by applying a channel transformation to the features and fusing them with legacy feature maps. These features, also known as logical features, include category attributes and other forms of semantic data. The feature map $N_{l}$ is calculated as Equations (3) and (4).

(1) $$M_{l}=\mathrm{CSP}\left(F_{l}\cup \mathrm{Upsample}\left(\mathrm{Transit}\left(M_{l+1}\right)\right)\right)\;\;l=3,4$$

(2) $$M_{5}=F_{5}$$

(3) $$N_{l}=\mathrm{CSP}\left(\mathrm{Transit}\left(M_{l}\right)\cup \mathrm{Downsample}\left(N_{l-1}\right)\right)\;\;l=4,5$$

(4) $$N_{3}=\mathrm{CSP}\left(M_{3}\right)$$

**Figure 3:**
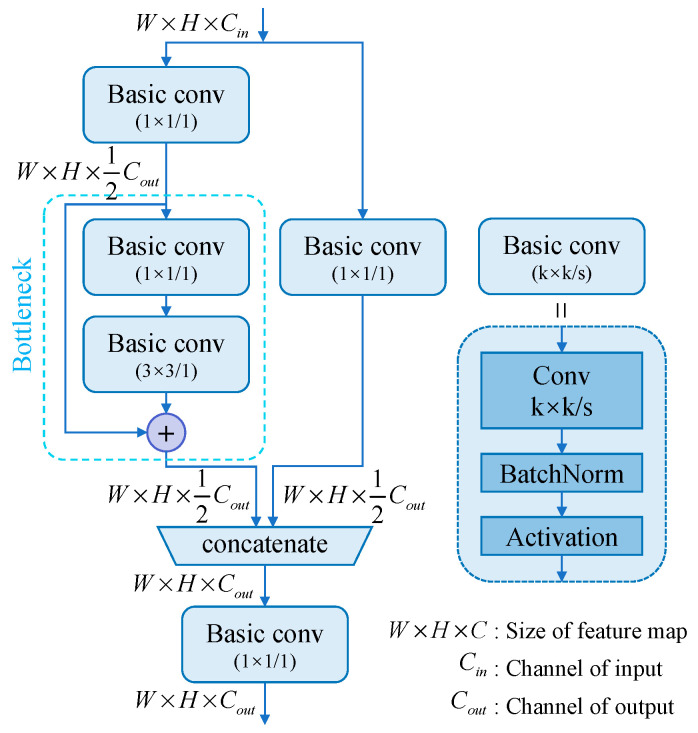
Pipeline of CSP Layer.

The pipeline for CSP(•), which depicts computation by the CSP layer [35], is depicted in Figure 3. Downsample(•) is a Basic conv(3 × 3/2) pipeline, while Upsample(•) is a nearest-sample interpolation. To make the change, Transit(•) uses Basic conv(3 × 3/1) to compress the channels of features. To obtain $M_{l}$, $F_{l}$ is sent to the CSP layer directly to preserve shallow information, and the information of deeper $M_{l+1}$ is refined by transition as a supplement. To obtain $N_{l}$, the $\mathrm{Transit}(M_{l})$, which is refined before with deep information, is concatenated with the deepened feature extracted from the shallower level.

In forward computing of the network, detailed shallow features close to the backbone network are provided for localization, and abstract deep information after multiple convolution calculations is provided for classification. Meanwhile, the goals of the two sub-tasks can focus on training certain components of the network independently, therefore increasing the network’s computational capacity.

### 3.2. Adaptive Core Prior

For the resulting map $O_{l}$ from the network at level l with the size of $W_{l}\times H_{l}\times (C+4+1)$, the anchor point on it can be denoted as $a_{l}=(x,y)$, where $x\in [1,W_{l}],y\in [1,H_{l}]$. $W_{l}$ and $H_{l}$ is the width and height of the map. The information at each anchor point includes C classification scores, four regression parameters, and an objectness score. The regression parameters can be denoted as $t_{x}$, $t_{y}$, $t_{w}$, and $t_{h}$. In the anchor-free mechanism, the predicted box $p_{\left(x,y\right)}^{box}$ of the anchor point is obtained with regression as follows:

(5) $$p_{\left(x,y\right)}^{box}=\{x_{c},y_{c},w_{\left(x,y\right)},h_{\left(x,y\right)}\}$$

(6) $$x_{c}=(x+t_{x})\cdot s_{l}$$

(7) $$y_{c}=(y+t_{y})\cdot s_{l}$$

(8) $$w_{\left(x,y\right)}=\exp(t_{w})\cdot s_{l}$$

(9) $$h_{\left(x,y\right)}=\exp(t_{h})\cdot s_{l}$$

where $p_{\left(x,y\right)}^{box}$ is represented by the coordinates of the center of the prediction box, and $x_{c}$ and $y_{c}$ with the width $w_{\left(x,y\right)}$ and height $h_{\left(x,y\right)}$ of the prediction box. $s_{l}$ indicates the scaling ratio of the map $O_{l}$ relative to the original image.

In order to alleviate the imbalance between positive and negative samples and enhance the stability of early training, the adaptive core prior is developed to perform the initial screening of anchor points. After framing the core area in the ground-truth (GT) box, the anchor points in the area and inside the GT box are marked as potential positive points, which are assigned lower training costs. On the contrary, the anchor points outside the core area or the GT box are assigned higher values of the training cost. The details of the procedure for cost will be elaborated in Section 3.3.

**Figure 4:**
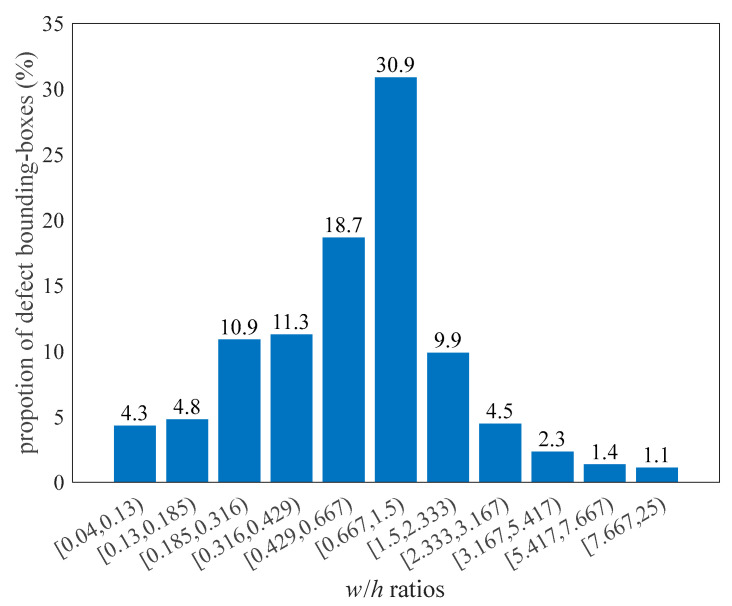
Distribution of ground-truth boxes’ aspect ratios in NEU-DET dataset.

Unlike objects in natural scenes, defects frequently appear in extreme shapes. Figure 4 depicts the aspect-ratio distribution of the GT boxes in the NEU-DET training set. It can be considered that when the aspect ratio is in the interval [0.667, 1.5), the bounding-box size of GT is close to 1:1, and the number of such GTs only accounts for 30.9%. A large proportion of bounding boxes are thin or flat. Therefore, instead of applying a fixed square center prior in the original YOLOX, we set the core area with corresponding ratios by calculating the aspect ratio of GT boxes, as shown in Equations (10) and (11). The width and height of the GT box are denoted as $w_{g}$ and $h_{g}$. The area of the core $A_{core}$ is set to 24, which is determined suitable by attempts.

(10) $$w_{core}=\sqrt{A_{core}\frac{w_{g}}{h_{g}}}$$

(11) $$h_{core}=\sqrt{A_{core}\frac{h_{g}}{w_{g}}}$$

**Figure 5:**
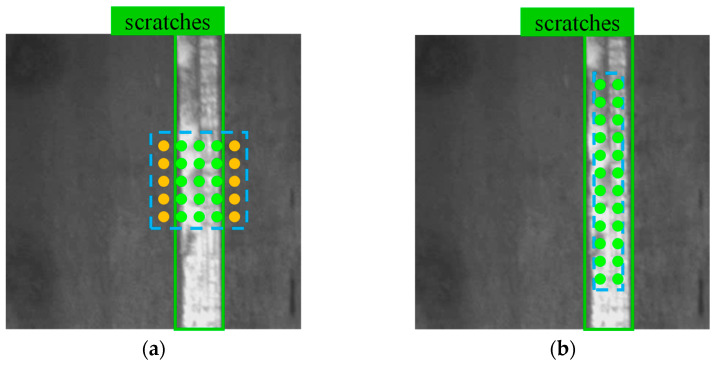
Groups of positive anchor points sampled by different core priors in the same ground-truth box. GT is indicated by a solid green line and the core areas are indicated by blue dashed lines. The anchor points within the core area are shown in the illustration, but only those in GT can be selected. The green points are finally selected as potential positive anchor points, while the yellow ones are abandoned. (**a**) Anchor points are sampled using multi positives method. (**b**) Anchor points are sampled with adaptive core prior.

This method is utilized to adapt the core area to GTs of different shapes, thus covering more potential positive anchor points than the original multi-positives method. As the example shown in Figure 5, for the anchor points $\left\{a\right\}$ generated from the result map, both methods select the points inside the core area and GT. As a result, fewer anchor points within the square center area are selected using the multi-positives method, which filters the yellow points outside the GT. However, the adaptive core prior can preserve more points both inside the core area and inside GT. Plenty of positives make the training more adequate. The method is integrated into the training process, which enables the model to learn to accommodate defects of different shapes in end-to-end training.

### 3.3. Self-Tuning Transport Assignment

Dense prediction produces many potential outcomes for each image; thus, it is important to cherry-pick the best training examples. In order to quantify the learning challenge posed by the training sample’s similarity to the goal label, the training cost was established. The number of training samples is too little and the quality of supervision is too low. If all samples are considered positive, then the optimization process will center on the many low-quality samples that will overwhelm the few high-quality ones.

Since the size and shape of each defect label affects the distribution of candidate samples that match to the GT, it is important to be able to divide samples flexibly into positive and negative categories. Meanwhile, it is important to pair each positive sample with an appropriate GT as its training goal. Therefore, a self-tuning transport assignment (STTA) is proposed based on the OTA [33] technique. A dynamic determination of the number of positive samples is made using the GT dimension, and the matching relationship between the sample set and the label set is then established.

Instead of gauging the relative cost difference between each GT, the SimOTA approach simply uses the total of IoU (the dynamic k) as the sample size for each GT. It leads to certain samples being wrongly classified into the k-positive samples, even though the gap between the two is substantial. The cost disparity between individuals in a sample is taken into account by STTA, in addition to the overall sample level. Incorporating the categorical and geographical quality of training samples reduces the overall training cost. Dynamic criteria can be used to filter out low-quality training samples, allowing you to fine-tune the network’s education.

## 4. Experiments

### 4.1. Datasets and Metrics

Our proposed model is evaluated, respectively, for two datasets, NEU-DET and GC10-DET.

NEU-DET [36] is a dataset of steel surfaces consisting of 1800 photos of hot-rolled steel strips. Crazing, inclusion, patches, pitted surface, rolled-in scales, and scratches are just some of the six types of flaws depicted in the photographs. It’s a 200 × 200 pixel image. A total of 1440 photos are used as the training set, while 360 are used as the test set in experimental settings.

The steel-sheet-surface dataset GC10-DET [37] contains 2292 annotated steel surface images, including punching hole (Pu), welding line (Wl), crescent gap (Cg), water spot (Ws), oil spot (Os), silk spot (Ss), inclusion (In), rolled pit (Rp), crease (Cr), and waste folding (Wf), with a total of 10 types of defects; the image size is 2048 × 1000.

To measure the precision of the model, we use the COCO metrics mAP [38]. The average precision (AP) is obtained by taking the integral of the P–R curve, which is obtained by computing the precision and recall of the detection findings at varying levels of confidence. The median AP is then calculated by averaging the APs for each category. The COCO criterion calculates mAP under a series of IoU thresholds (IoU thresholds from 0.5 to 0.95 with steps of 0.05) and takes the average value, denoted as mAP_@[0.5:0.95]_; this indicator is more comprehensive. In addition, when mAP is calculated under the IoU threshold of 0.5, it is denoted as mAP_@0.5_, which is commonly used in defect detection.

Frames per second (FPS) is used to measure how quickly a model can process an image, and the inference time of the model for a single image (including NMS) is used to determine how many frames per second contain recognizable images.

### 4.2. Implementation Details

The implementation settings of our model ST-YOLO are set as follows. Since the downsampling factor of our model is 32, the input size is set to 224 × 224 for the NEU-DET dataset, and it is set to 512 × 256 for the GC10-DET dataset. Before training a model, we load the CSPDarkNet-53 weights that have already been trained on ImageNet into the backbone network. In the first phase, the core network is frozen, and in the second, it is thawed. We’ve decided on a batch size of 16. In the freezing phase, the starting learning rate is 0.001, and the training period is 150 epochs. It takes 100 epochs to complete training after an initial learning rate of 0.0001. The Adam optimizer is utilized, and the training is stabilized by a warm-up for the learning rate. With a ratio of 0.97 each epoch, the exponential decay is applied to the learning rate schedule.

The same strategy settings of training are set for other methods, including weights of backbone network pre-trained on ImageNet, two-stage training for frozen and unfrozen, and the warm-up and decay schedule. But due to the various computation of different models, the batch size needs to be adjusted accordingly, and the initial learning rate scales accordingly. The termination condition of training is set as the accuracy of the test set converges to a standard deviation of less than 0.005 mAP_@0.5_ every 10 epochs and a standard deviation of less than 0.001 mAP_@[0.5, 0.95]_ every 10 epochs.

The GeForce RTX3060 GPU serves as the experimental platform for all the tests. One FPS is measured at a time on the test set.

### 4.3. Experiment Results

**Figure 6:**
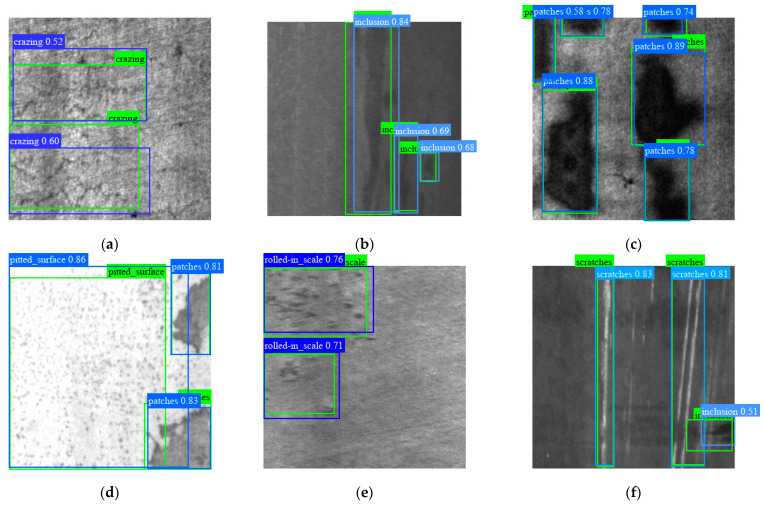
Defect-detection results on NEU-DET. The blue boxes represent the prediction results from our model, and the ground-truth labels of defects are indicated by the green boxes.

S-YOLO refers to the SFN-structured model, and ST-YOLO refers to the S-YOLO model trained with STTA. Table 1 and Table 2 show the mAP_@0.5_ and AP_@0.5_ of each category, respectively, based on experimental findings of the proposed model for the defects on the test set (with the evaluation threshold set to IoU = 0.5). The photos in Figure 5 and Figure 6 are a few instances of those discovered by ST-YOLO. The blue boxes indicate the model’s predictions, while the tags in the top left corner illustrate the types of flaws and the confidence with which the model predicts that they exist. The defect’s ground-truth label is displayed in the green box, and the defect kind is shown in the upper right.

When put to the test on the NEU-DET dataset, the S-YOLO model with SFN outperforms the baseline YOLOX by 2.2 mAP_@0.5_. Based on these results, ST-YOLO trained by STTA achieves 80.3 mAP_@0.5_, which is an improvement of 3.2 mAP_@0.5_ over the baseline. Table 1 shows significant progress in the detection of tough discrete-type flaws such as crazing and rolled-in scale.

As shown in Figure 6, the detector can correctly identify the defect category. In an environment with poor lighting and low contrast, the detector still has good detection ability for the collected images, as illustrated in Figure 6b,e. As can be seen in Figure 6a,e, the shunt fusion structure allows the model to accurately distinguish and characterize defect locations in the presence of discretely distributed defect groups. Small faults are also easily identifiable using the detector, as shown in Figure 6b,c. Figure 6b,f demonstrates how the adaptive core prior may adjust the bounding box so that it perfectly encloses irregularly shaped defects like inclusions and scratches. In some cases where multiple types of defects coexist or even overlap, the algorithm is still competent, as depicted in Figure 6d,f.

In the experiment of the GC10-DET dataset, compared with the baseline YOLOX, the SFN improves our model by 1.5 mAP_@0.5_. After training with the self-tuning TA method, ST-YOLO can further improve by 1.2 mAP_@0.5_ to 72.8. In terms of category shown in Table 2, the shunt fusion network architecture resulted in a large improvement in the detection of discrete defects like silk spot. The adaptive core prior has stronger adaptability to long-shaped defects (such as welding lines), and it makes the localization more accurate.

**Figure 7:**
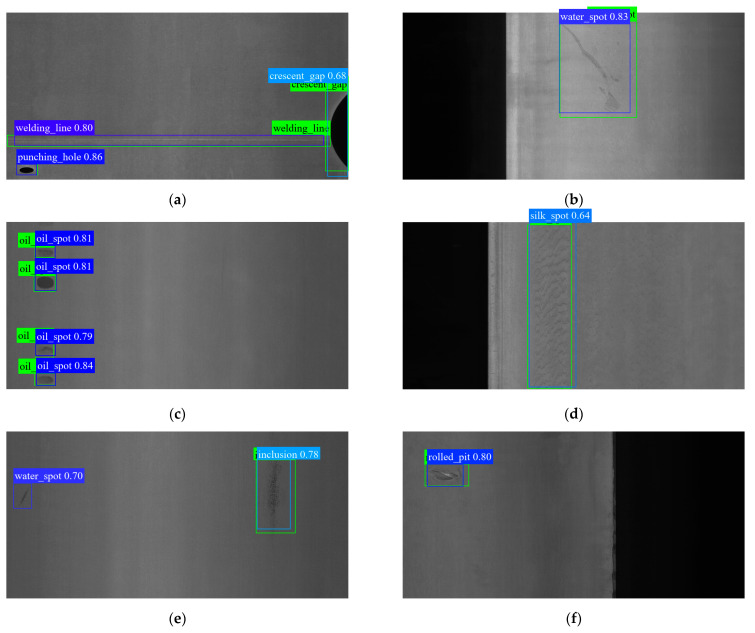

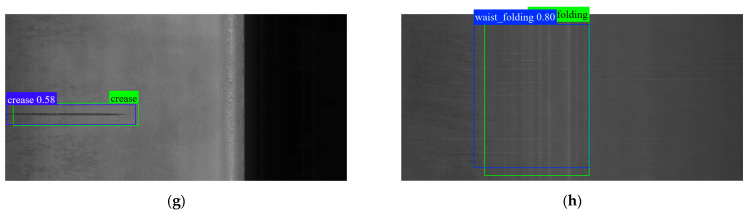
(**a**–**h**) Examples of defect-detection results on GC10-DET.

It can be found that the model can accurately locate defects of a small size and extreme aspect ratio (such as punching holes, oil spots, and welding lines), from Figure 7a,c. For speckled-texture-type defects (such as silk spot and inclusion), the model shows promising feature-extraction ability, as shown in the results in Figure 7d,e. Although some non-salient defects are blurred with the background, the model is robust and can detect defects (such as water spots and waist folding), as shown in Figure 7b,h. It is worth noting that the detector can still detect small defects omitted in the annotations of datasets, such as the water spot on the left side of Figure 7e.

### 4.4. Comparison with the State-of-the-Art Methods

To test the performance of the model more comprehensively, the proposed model is compared with the existing mainstream detection models in terms of accuracy, inference speed, and parameter scale. The compared models involve anchor-based detectors (including Faster R-CNN, RetinaNet, and YOLOv4) and anchor-free detectors (including FCOS, CenterNet, and YOLOX). The results of different methods on the same test set are shown in Table 3 and Table 4.

The proposed ST-YOLO achieves the highest accuracy, with steady improvement over the baseline on both NEU-DET and GC10-DET datasets, similar to YOLOX in speed and scale. In addition, STTA is only performed during training and does not affect the inference speed and parameter scale of the model.

From the perspective of anchor manner, the anchor-free methods generally outperform the anchor-based methods in terms of accuracy and speed. Because of a large variation in the shape and size of defects, the pre-defined anchors perform poor fitting ability to the data when regressing to calculate the bounding box. As a result, the flexible anchor-free mechanism has a greater advantage in accuracy. Furthermore, due to the large number of predicted boxes generated by the anchor-based methods, which brings a certain computational burden, the methods are inferior in speed.

Among the anchor-based detectors, Faster R-CNN (with FPN) reaches the highest mAP due to the architecture of the two-stage network which fine-tunes the candidates in the second stage. But also, because of the second calculation, the speed is slower in contrast with the one-stage model (RetinaNet and YOLOv4). Meanwhile, YOLOv4 adopts clustering to optimize the size of anchor templates on the training set to suit the data distribution of defects. Therefore, benefiting from the effect of the backbone and the optimization of anchors, the mAP of YOLOv4 is higher than that of RetinaNet. Among the three, RetinaNet showed the largest drop (22% mAP_@0.5_) in accuracy in the GC10-DET dataset, which differs in the size of defects.

Among the anchor-free detectors, the structure of FCOS is most similar to that of RetinaNet. The difference between the two lies in the label assignment method except for the anchor mechanism. FCOS employs spatial constraints and scales constraints to assign labels, instead of the fixed IoU threshold utilized in RetinaNet. Both factors increase the speed and accuracy of the FCOS. Among the detectors, only the CenterNet calculation results are performed on a single-scale feature map instead of the multi-scale feature maps, so the speed is the fastest. However, the robustness of the single-scale feature map is poor when testing on the GC10-DET.

In the YOLO series of detectors, compared with YOLOv4, YOLOX replaces the anchor mechanism in an anchor-free manner, and replaces the sibling head with decoupled heads, and adopts the dynamic label assignment method SimOTA. As a result, YOLOX has improved speed and accuracy compared to YOLOv4. Based on YOLOX, our model ST-YOLO further extends the feature decoupling to the stage of feature fusion, and employs the more flexible label assignment in a self-tuning manner, to achieve stable and outstanding accuracy with little additional computation and parameters.

In addition to comparing with some specific classic defect-recognition techniques mentioned above, we also compare ST-YOLO with related research, including Li’s Optimized-Inception-ResnetV2 [39], Chen’s FRCN [40], Cheng’s DE_RetinaNet [16], Liu’s RAF-SSD [41], Tang’s ECA+MSMP [42], and improved YOLOX with boosting [43]. The detailed comparison results are recorded in Table 5. Of course, compared to related research, ST-YOLO still achieves the dual advantages of recognition accuracy and speed. Sufficient comparative experiments have demonstrated that the proposed ST-YOLO defect-recognition framework has strong capabilities and advantages.

## 5. Discussion and Analysis

### 5.1. Ablation Studies

In order to verify the effectiveness of the proposed network structure and training method for detection, different feature fusion networks and different label assignment strategies are compared and tested. The implementation settings can be found in Section 4.2.

#### 5.1.1. Shunt Fusion Network Structure

The feature network fuses features from different depths of the backbone network to provide feature maps with different receptive fields for the head. In this section, five feature fusion networks with different structures are compared with the original YOLOX feature fusion network, to explore the optimal feature fusion method. The tested models adopt the same backbone CSPDarkNet-53 and the same training strategy SimOTA, and the training parameters are consistent as well. The test results on the test set of the NEU-DET dataset are listed in Table 6.

**Figure 8:**
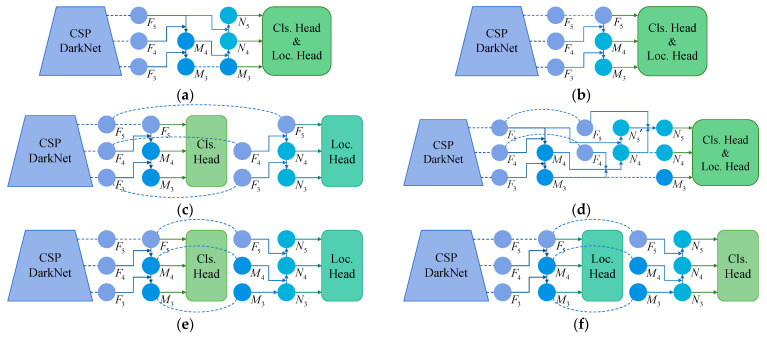
Structures of different fusion flow for feature fusion networks: (**a**) FPN-PAN, (**b**) single FPN, (**c**) double FPN, (**d**) BTFPN, (**e**) S-Cls-Loc, and (**f**) S-Loc-Cls.

A top-down and bottom-up bi-directional fusion flow is adopted in the original YOLOX, and the structure is FPN-PAN. This structure can facilitate information sharing between deep and shallow features. Its structure is shown in Figure 8a, and mAP_@0.5_ is only 77.1. When the single top-down fusion flow is applied and the structure is single FPN, as shown in Figure 8b, mAP_@0.5_ has been improved slightly. This suggests that some valid information may be submerged in the late PAN structure. In the structure of Figure 8c, when the fusion process for classification and localization are separated by two parallel FPNs, the structure is double FPN, whose mAP_@0.5_ reaches 78.7. It is demonstrated that the feature fusion of the shunted way helps to improve the detection of the model.

In our proposed SFN structure, which can also be called S-Loc-Cls, the localization features are first fused from the top down, and then the classification features are fused from the bottom up, as illustrated in Figure 8f. Furthermore, the inverse structure of SFN is designed, which is named S-Cls-Loc shown in Figure 8e, and its mAP_@0.5_ is inferior to our method. It shows that the features in the early stage are more helpful for localization learning, while the features fused in the later stage are more helpful for classification learning. To verify the superiority of SFN in YOLOX, the recent method proposed in [44], the BTFPN, is also compared and is shown in Figure 8d. Summarizing the above comparisons, our method achieves the highest 79.3 mAP_@0.5_, as the best structure in feature fusion.

In essence, from the perspective of the calculation course, in Figure 8a, as the receptive field expands with the convolution stack, the feature information deepens layer by layer from the bottom to the top, and gradually deepens from left to right. The $F_{l}$ extracted by the backbone network contains much position information, which needs to be captured by the localization head in time. In the fusion process, convolution computing further abstracts the feature information, to gain features related to the category and input it into the classification head. As depicted in Table 6, when the localization head is fed with shallow features, such as the structure of Figure 8b,c,f, the accuracy of discrete defects (such as crazing, pitted surface, and rolled-in scale) have improved. Because the distinctive pixels of these defects are discontinuous and small, the deep convolutions drown out the detailed position information. However, the features of these categories, which are represented by the combination of distinctive pixel groups, require deep extraction. By contrast, for continuous defects (such as patches and scratches), the depth of feature maps processed by the head has little influence on the detection. Because such defects appear as simply connected spaces in the image, feature extraction is relatively easy and concentrated.

Therefore, the computational characteristics of the proposed SFN conform to the feature depth of defects, and the structure performs better on detection.

#### 5.1.2. Self-Tuning Label Assignment for Training

A suitable label assignment strategy is very important because the quality of the samples affects the training of the detector. In order to measure the quality of the samples, for the positive sample set {*p*} finally matched to the GT *g*, the quality score of the positive sample set is defined as $Q_{g,\{p\}}$:

(12) $$Q_{g,\{p\}}=\exp\left(-\frac{1}{\min\left\{c_{g,\{p\}}\right\}}\left(\mathrm{mean}\left\{c_{g,\{p\}}\right\}-\min\left\{c_{g,\{p\}}\right\}\right)\right)$$

where $\left\{c_{g,\{p\}}\right\}$ represents the costs of positive samples {*p*}. The $\min\left\{c_{g,\{p\}}\right\}$ represents the minimum cost of positive samples matched to *g*, which also means the cost of the best sample. The overall quality of the selected positive samples is evaluated by comparing the difference between the average cost of the positive samples and the cost of the best samples. The closer $Q_{g,\{p\}}$ is to 1, the higher the quality of the sample set.

**Figure 9:**
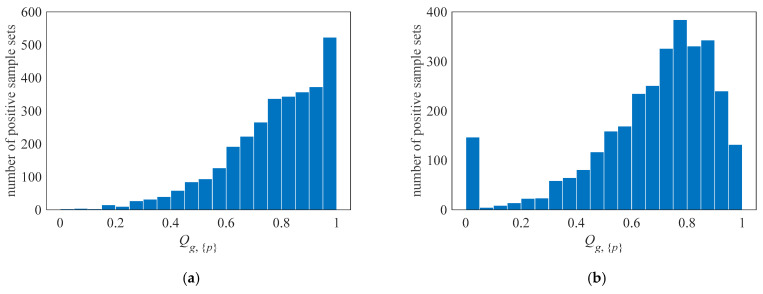
Distribution of quality scores of positive sample sets: (**a**) samples trained by STTA, and (**b**) samples trained by SimOTA.

During the training process of NEU-DET, the quality scores of the positive sample set corresponding to each GT are counted, and their distribution is illustrated in Figure 9. When self-tuning TA is applied for training, the overall quality of positive samples is high, and the quality scores of the sample sets matched by most of the GTs are close to 1. When training by SimOTA, the quality scores of most positive sample sets are concentrated around 0.8, and there are some sample sets whose scores are seriously low. SimOTA only considers the relative rank of the training cost and does not consider the value of the training cost *c* itself, which leads to some low-quality samples with large gaps as positive samples. However, STTA adjusts the concerned anchors through the adaptive core prior, and then discards deteriorating samples and adds tolerance bands. The method corrects the screening threshold for training costs, thereby improving the quality of the positive sample set.

Further, in order to test the robustness of STTA, it is employed for training on the network structures of YOLOX and S-YOLO, and evaluated on the test set of NEU-DET and GC10-DET, as shown in Table 7. It can be found that compared with the model using SimOTA, STTA can stably improve the model performance and the extra time consumed for training is negligible.

### 5.2. Limitations and Future Works

Although our model ST-YOLO has made good progress in testing, there are still some limitations to be improved. First, the calculation amounts of the two parallel branches after splitting in SFN are different, which leads to a part of the computing time being wasted during model operation. Therefore, it is necessary to balance the computational load between the two branches to optimize the computational efficiency of the network. Second, the image form of discrete defects is special, and it is helpful to explore the impact of features extracted with convolutional networks at different semantic levels on detection. In addition, the STTA still has some hyperparameters to be set. In the future, the calculation of descriptive statistics will replace the hyperparameters used for sample screening, to adaptively determine the number of primary screening samples according to the distribution of the samples. In addition, how to achieve efficient information flow collaboration between cameras and image data directly, as well as information exchange on industrial sites, are also key issues that need to be addressed to truly implement defect-monitoring technology in the field, which is also our future research focus [45,46].

## 6. Conclusions

In this work, we present the ST-YOLO model for identifying surface defects in steel. Discrete defect identification has been enhanced thanks to an architecture that uses a shunt feature fusion network to optimize the calculation process for defect classification and localization. Our model’s learning and fitting abilities are enhanced by the algorithm’s flexibility to account for the dynamic nature of the data distribution of faults and its use of dynamic training criteria to choose samples of high quality for training. Our model scores 80.3 mAP on the NEU-DET dataset, while running at 46.0 FPS in the experiment, and 72.8 mAP, while running at 44.7 FPS in the GC10-DET dataset. Accuracy is enhanced, and overall performance is exceptional compared to the baseline. The mechanism of the proposed methods is illustrated by contrast in experiments and analysis from the discussion.

In future work, we will continue to optimize the structure of the network from the perspective of computational efficiency, and further explore the relationship between the semantic level of defect images and the mechanics of the detection network. In addition, developing a training strategy with less hyperparameter intervention is also a direction for future research.

## Figures and Tables

**Table 1:** Results of detection on NEU-DET.

Model	mAP_@0.5_	Crazing	Inclusion	Patches	Pitted Surface	Rolled-in Scale	Scratches
YOLOX	77.1	46.6	83.1	88.6	83.5	64.8	95.7
S-YOLO	79.3	48.8	**83.2**	**90.7**	**87.4**	69.9	96.0
ST-YOLO	**80.3**	**54.6**	83.0	89.2	84.7	**73.2**	**97.0**

**Table 2:** Results of detection on GC10-DET.

Model	mAP_@0.5_	Pu	Wl	Cg	Ws	Os	Ss	In	Rp	Cr	Wf
YOLOX	70.1	91.9	92.0	99.2	72.9	72.1	54.7	36.6	44.4	60.4	77.0
S-YOLO	71.6	87.1	92.1	99.6	**75.3**	69.7	58.2	36.0	**49.0**	**74.5**	74.5
ST-YOLO	**72.8**	**93.4**	**97.3**	**99.6**	73.5	**72.5**	**59.3**	**37.9**	44.6	70.5	**79.7**

**Table 3:** Performance comparison results on NEU-DET.

Model	Backbone	mAP_@[0.5:0.95]_ (%)	mAP_@0.5_(%)	F1 Score(%)	FPS	Params
Faster-RCNN w FPN	ResNet-50	38.5	76.5	72	28.7	41.38 M
RetinaNet	ResNet-50	34.8	69.6	67	42.6	36.43 M
YOLOv4	CSPDarkNet-53	35.8	76.2	72	40.3	63.96 M
FCOS	ResNet-50	40.1	77.5	73	45.9	**32.13 M**
CenterNet	ResNet-50	38.5	75.1	71	**78.3**	32.66 M
YOLOX	CSPDarkNet-53	40.3	77.1	74	48.9	54.15 M
YOLOX w boosting	CSPDarkNet-53	40.9	78.2	74	48.7	57.96 M
S-YOLO (Ours)	CSPDarkNet-53	40.8	79.3	76	46.0	55.82 M
ST-YOLO (Ours)	CSPDarkNet-53	**40.9**	**80.3**	**78**	46.0	55.82 M

**Table 4:** Performance comparison results on GC10-DET.

Model	Backbone	mAP_@[0.5:0.95]_ (%)	mAP_@0.5_(%)	F1 Score(%)	FPS	Params
Faster-RCNN w FPN	ResNet-50	32.7	67.4	65	26.1	41.40 M
RetinaNet	ResNet-50	25.7	54.4	56	39.3	36.52 M
YOLOv4	CSPDarkNet-53	28.0	67.0	65	41.0	63.99 M
FCOS	ResNet-50	32.0	69.3	67	41.8	**32.14 M**
CenterNet	ResNet-50	29.1	63.8	62	**68.8**	32.66 M
YOLOX	CSPDarkNet-53	31.4	70.1	69	48.5	54.15 M
S-YOLO (Ours)	CSPDarkNet-53	32.0	71.6	68	44.7	55.83 M
ST-YOLO (Ours)	CSPDarkNet-53	**32.9**	**72.8**	**71**	44.7	55.83 M

**Table 5:** Performance comparison results on NEU-DET with some related researches.

Related Researches	mAP_@0.5_ (%)	FPS
Li’s Optimized-Inception-ResnetV2 [39]	78.1	24.0
Chen’s FRCN [40]	77.9	27.5
Cheng’s DE_RetinaNet [16]	78.25	30.0
Liu’s RAF-SSD [41]	75.1	35.5
Tang’s ECA+MSMP [42]	**80.86**	27.9
YOLOX w boosting [43]	78.2	40.7
S-YOLO (Ours)	79.3	**46.0**
ST-YOLO (Ours)	80.3	**46.0**

**Table 6:** Accuracy of different fusion structures in the neck of YOLOX model.

Fusion Structures	mAP_@0.5_ on NEU-DET	Crazing	Inclusion	Patches	Pitted Surface	Rolled-in Scale	Scratches
FPN-PAN	77.1	46.6	83.1	88.6	83.5	64.8	95.7
single FPN	77.9	48.9	83.2	88.6	84.9	66.0	95.9
double FPN	78.7	48.8	82.2	91.0	**87.8**	66.3	**96.2**
BTFPN	78.1	**49.7**	81.2	90.4	86.6	65.9	94.6
S-Cls-Loc	77.2	44.1	82.6	**91.4**	84.8	64.6	95.4
S-Loc-Cls (Ours)	**79.3**	48.8	**83.2**	90.7	87.4	**69.9**	96.0

**Table 7:** The influence of different label assignment methods on model accuracy.

Model Structure	Label Assignment Method	mAP_@0.5_ on NEU-DET	FPS in Training on NEU-DET	mAP_@0.5_ on GC10-DET	FPS in Training on GC10-DET
YOLOX	SimOTA	77.1	**53.7**	70.1	**47.9**
YOLOX	**STTA**	**77.9**	49.6	**71.9**	44.5
S-YOLO	SimOTA	79.3	**51.2**	71.6	**46.3**
S-YOLO	**STTA**	**80.3**	47.7	**72.8**	43.0

## Data Availability

Data are contained within the article.

## References

[1] Song G., Song K., Yan Y. (2020). EDRNet: Encoder-Decoder Residual Network for Salient Object Detection of Strip Steel Surface Defects. IEEE Trans. Instrum. Meas..

[2] Ahmad H.M., Rahimi A. (2022). Deep learning methods for object detection in smart manufacturing: A survey. J. Manuf. Syst..

[3] Tao X., Hou W., Xu D. (2021). A Survey of Surface Defect Detection Methods Based on Deep Learning. Acta Autom. Sin..

[4] Chu M., Gong R., Gao S., Zhao J. (2017). Steel surface defects recognition based on multi-type statistical features and enhanced twin support vector machine. Chemom. Intell. Lab. Syst..

[5] Wang J., Li Q., Gan J., Yu H., Yang X. (2020). Surface defect detection via entity sparsity pursuit with intrinsic priors. IEEE Trans. Industr. Inform..

[6] Liu X., Xu K., Zhou P., Zhou D., Zhou Y. (2020). Surface defect identification of aluminium strips with non-subsampled shearlet transform. Opt. Lasers Eng..

[7] Yu H., Li Q., Tan Y., Gan J., Wang J., Geng Y.-A., Jia L. (2019). A Coarse-to-Fine Model for Rail Surface Defect Detection. IEEE Trans. Instrum. Meas..

[8] Zhang J., Wang H., Tian Y., Liu K. (2020). An accurate fuzzy measure-based detection method for various types of defects on strip steel surfaces. Comput. Ind..

[9] Liu K., Wang H., Chen H., Qu E., Tian Y., Sun H. (2017). Steel Surface Defect Detection Using a New Haar–Weibull-Variance Model in Unsupervised Manner. IEEE Trans. Instrum. Meas..

[10] Gao Y., Li X., Wang X.V., Wang L., Gao L. (2022). A Review on Recent Advances in Vision-based Defect Recognition towards Industrial Intelligence. J. Manuf. Syst..

[11] Tulbure A.-A., Dulf E.-H. (2022). A review on modern defect detection models using DCNNs—Deep convolutional neural networks. J. Adv. Res..

[12] Usamentiaga R., Lema D.G., Pedrayes O.D., Garcia D.F. (2022). Automated Surface Defect Detection in Metals: A Comparative Review of Object Detection and Semantic Segmentation Using Deep Learning. IEEE Trans. Ind. Appl..

[13] Zhou T., Zhang J., Su H., Zou W., Zhang B. (2021). EDDs: A series of Efficient Defect Detectors for fabric quality inspection. Measurement.

[14] Ge Z., Liu S., Wang F., Li Z., Sun J. (2021). YOLOX: Exceeding YOLO Series in 2021. http://arxiv.org/abs/2107.08430.

[15] Liu Z., Tang R., Duan G., Tan J. (2021). TruingDet: Towards high-quality visual automatic defect inspection for mental surface. Opt. Lasers Eng..

[16] Cheng X., Yu J. (2021). RetinaNet with Difference Channel Attention and Adaptively Spatial Feature Fusion for Steel Surface Defect Detection. IEEE Trans. Instrum. Meas..

[17] Ma Z., Li Y., Huang M., Huang Q., Cheng J., Tang S. (2022). A lightweight detector based on attention mechanism for aluminum strip surface defect detection. Comput. Ind..

[18] Kou X., Liu S., Cheng K., Qian Y. (2021). Development of a YOLO-V3-based model for detecting defects on steel strip surface. Measurement.

[19] Tian R., Jia M. (2022). DCC-CenterNet: A rapid detection method for steel surface defects. Measurement.

[20] Yu J., Cheng X., Li Q. (2022). Surface Defect Detection of Steel Strips Based on Anchor-Free Network With Channel Attention and Bidirectional Feature Fusion. IEEE Trans. Instrum. Meas..

[21] Oksuz K., Cam B.C., Kalkan S., Akbas E. (2021). Imbalance Problems in Object Detection: A Review. IEEE Trans. Pattern Anal. Mach. Intell..

[22] Ren S., He K., Girshick R., Sun J. Faster R-CNN: Towards Real-Time Object Detection with Region Proposal Networks. Proceedings of the Advances in Neural Information Processing Systems 28 (NIPS 2015).

[23] Dai J., Qi H., Xiong Y., Li Y., Zhang G., Hu H., Wei Y. Deformable convolutional networks. Proceedings of the 2017 IEEE International Conference on Computer Vision (ICCV).

[24] Redmon J., Farhadi A. YOLOv3: An Incremental Improvement. https://arxiv.org/abs/1804.02767.

[25] Song G., Liu Y., Wang X. Revisiting the Sibling Head in Object Detector. Proceedings of the IEEE Computer Society Conference on Computer Vision and Pattern Recognition.

[26] Wu Y., Chen Y., Yuan L., Liu Z., Wang L., Li H., Fu Y. Rethinking Classification and Localization for Object Detection. Proceedings of the IEEE Computer Society Conference on Computer Vision and Pattern Recognition.

[27] Feng C., Zhong Y., Gao Y., Scott M.R., Huang W. TOOD: Task-aligned One-stage Object Detection. Proceedings of the IEEE International Conference on Computer Vision.

[28] Tian Z., Shen C., Chen H., He T. (2020). FCOS: A simple and strong anchor-free object detector. arXiv.

[29] Kong T., Sun F., Liu H., Jiang Y., Li L., Shi J. (2020). FoveaBox: Beyound Anchor-Based Object Detection. IEEE Trans. Image Process.

[30] Zhang S., Chi C., Yao Y., Lei Z., Li S.Z. Bridging the gap between anchor-based and anchor-free detection via adaptive training sample selection. Proceedings of the IEEE/CVF Conference on Computer Vision and Pattern Recognition.

[31] Lin T.-Y., Goyal P., Girshick R., He K., Dollár P. (2017). Focal Loss for Dense Object Detection. arXiv.

[32] Kim K., Lee H.S. (2020). Probabilistic Anchor Assignment with IoU Prediction for Object Detection. Lecture Notes in Computer Science (Including Subseries Lecture Notes in Artificial Intelligence and Lecture Notes in Bioinformatics).

[33] Ge Z., Liu S., Li Z., Yoshie O., Sun J. OTA: Optimal Transport Assignment for Object Detection. Proceedings of the IEEE Computer Society Conference on Computer Vision and Pattern Recognition.

[34] Bochkovskiy A., Wang C.-Y., Liao H.-Y.M. (2020). YOLOv4: Optimal Speed and Accuracy of Object Detection. arXiv.

[35] Wang C.Y., Liao H.Y.M., Wu Y.H., Chen P.Y., Hsieh J.W., Yeh I.H. CSPNet: A new backbone that can enhance learning capability of CNN. Proceedings of the IEEE Computer Society Conference on Computer Vision and Pattern Recognition Workshops.

[36] He Y., Song K., Meng Q., Yan Y. (2020). An End-to-End Steel Surface Defect Detection Approach via Fusing Multiple Hierarchical Features. IEEE Trans. Instrum. Meas..

[37] Lv X., Duan F., Jiang J.-J., Fu X., Gan L. (2020). Deep Metallic Surface Defect Detection: The New Benchmark and Detection Network. Sensors.

[38] Padilla R., Netto S.L., da Silva E.A.B. A Survey on Performance Metrics for Object-Detection Algorithms. Proceedings of the International Conference on Systems, Signals, and Image Processing.

[39] Li Z., Tian X., Liu X., Liu Y., Shi X. (2022). A Two-Stage Industrial Defect Detection Framework Based on Improved-YOLOv5 and Optimized-Inception-ResnetV2 Models. Appl. Sci..

[40] Chen X., Lv J., Fang Y., Du S. (2022). Online Detection of Surface Defects Based on Improved YOLOV3. Sensors.

[41] Liu X., Gao J. (2021). Surface Defect Detection Method of Hot Rolling Strip Based on Improved SSD Model. Lecture Notes in Computer Science (Including Subseries Lecture Notes in Artificial Intelligence and Lecture Notes in Bioinformatics).

[42] Tang M., Li Y., Yao W., Hou L., Sun Q., Chen J. (2021). A strip steel surface defect detection method based on attention mechanism and multi-scale maxpooling. Meas. Sci. Technol..

[43] Bustillo A., Urbikain G., Perez J.M., Pereira O.M., de Lacalle L.N.L. (2018). Smart optimization of a friction-drilling process based on boosting ensembles. J. Manuf. Syst..

[44] Wang G., Liu Z., Sun H., Zhu C., Yang Z. (2022). Yolox-BTFPN: An anchor-free conveyor belt damage detector with a biased feature extraction network. Measurement.

[45] Tapia E., Sastoque-Pinilla L., Lopez-Novoa U., Bediaga I., de Lacalle N.L. (2023). Assessing Industrial Communication Protocols to Bridge the Gap between Machine Tools and Software Monitoring. Sensors.

[46] Fernández B., González B., Artola G., De Lacalle N.L., Angulo C. (2019). A Quick Cycle Time Sensitivity Analysis of Boron Steel Hot Stamping. Metals.

